# Supplementary material for: The causal relationship between the human gut microbiota and pyogenic arthritis: a Mendelian randomization study
Source: Front Cell Infect Microbiol. 2024 Nov 26;14:1452480. doi: 10.3389/fcimb.2024.1452480 (PMC11629706; doi:10.3389/fcimb.2024.1452480)
Supplement: Supplementary file 1 [file DataSheet1.zip › mendelian test/ebi-a-GCST90027546.csv_leave_one_out.pdf]

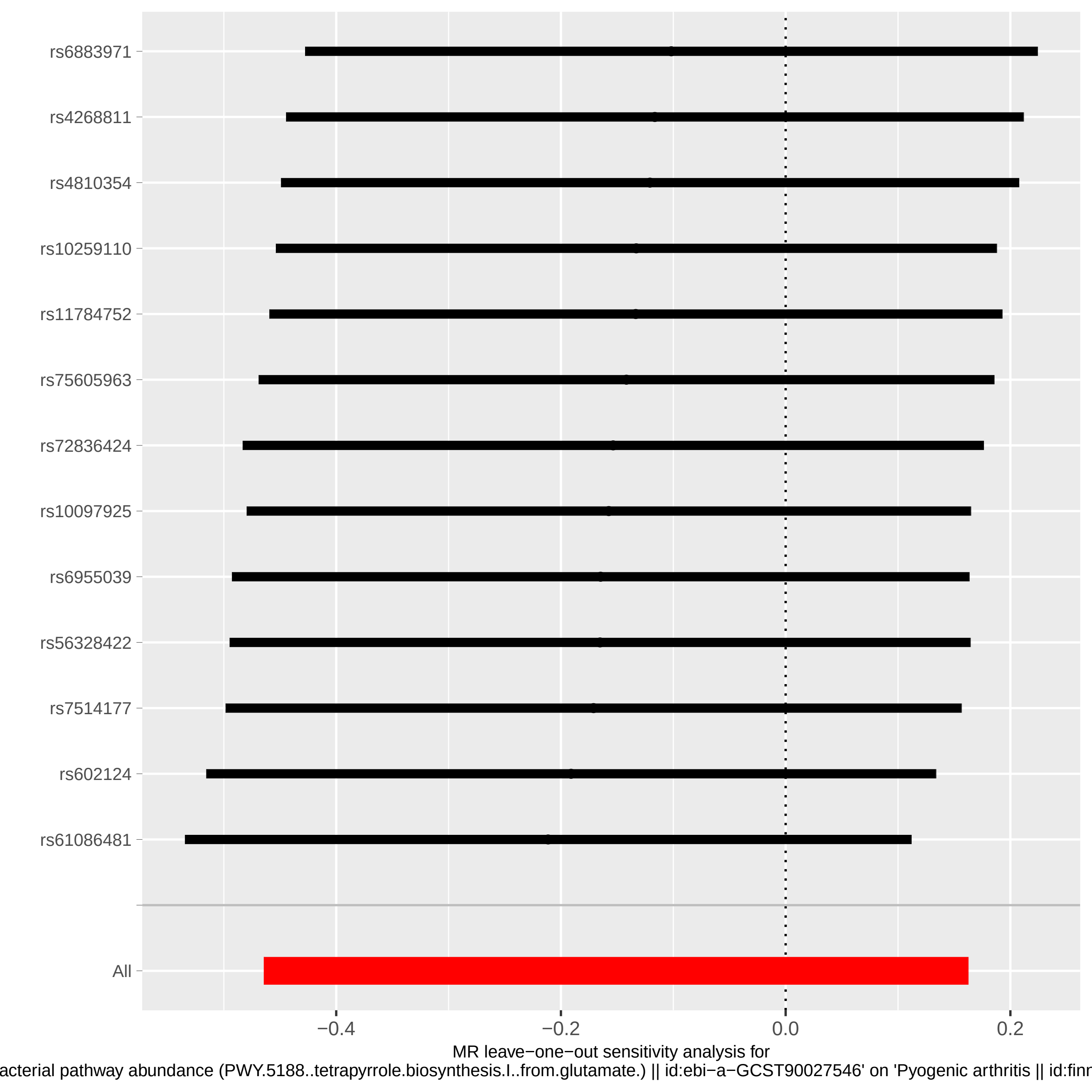
rs6883971
rs4268811
rs4810354
rs10259110
rs11784752
rs75605963
rs72836424
rs10097925
rs6955039
rs56328422
rs7514177
rs602124
rs61086481
All
−0.4
−0.2
0.0
0.2
MR leave−one−out sensitivity analysis for
acterial pathway abundance (PWY.5188..tetrapyrrole.biosynthesis.I..from.glutamate.) || id:ebi−a−GCST90027546' on 'Pyogenic arthritis || id:finn
